# The Attentional Orientation and Disengagement Mechanisms in Non-Target Processing of Self-Related Information and the Modulating Effect of Attentional Window

**DOI:** 10.3390/bs16081354

**Published:** 2026-08-06

**Authors:** Pengcheng Zhang, Zhiyi Huang, Boyan Jiang, Xiangping Gao

**Affiliations:** 1 Department of Psychology, School of Education, Zhejiang International Studies University, Hangzhou 310023, China; 2 Department of Psychology, Shanghai Normal University, Shanghai 200234, China

**Keywords:** self-relevant information, attentional orienting, attentional disengagement, attentional window

## Abstract

Whether the attentional capture mechanism of self-relevant information acting as non-target processing is entirely automatic or can be modulated by top-down goal setting remains inconclusive. Additionally, research has found that the attentional window modulates cognitive processing. Based on this, the present study employs a color-identity association paradigm and a modified spatial cueing paradigm to investigate the attentional capture mechanism (including attentional orienting and disengagement) of self-relevant information during non-target processing, as well as the moderating effect of the attentional window, through three experiments. Under the small attentional window condition, when self-relevant information was target-relevant (Experiment 1), participants showed faster attentional orienting and delayed disengagement toward self-relevant information compared to stranger information. When self-relevant information was target-irrelevant (Experiment 2), there were no significant differences in attentional orienting or disengagement between self-relevant and stranger information. Under the large attentional window condition, when self-relevant information was target-irrelevant (Experiment 3), participants exhibited faster attentional orienting and delayed disengagement toward self-relevant information compared to stranger information. The results of Experiments 1 and 2 demonstrate that attentional capture during non-target processing of self-relevant information is not entirely automatic but is modulated by top-down goal setting. The findings from Experiments 2 and 3 indicate that attentional window size regulates the mechanisms of attentional orienting and disengagement for self-relevant information during non-target processing. Collectively, these results reveal that although self-relevant information possesses marked salience, its processing remains subject to modulation by top-down goal setting. Furthermore, external factors such as attentional window size influence individual processing patterns of self-relevant information, demonstrating cognitive flexibility in self-relevant processing. This reflects individuals’ capacity for adaptive adjustments to environmental changes.

## 1. Introduction

### 1.1. The Self-Prioritization Effect in Information Processing

When stimuli are associated with self-concept, the stronger this association, the faster their processing and the better the memory performance they elicit. This phenomenon is referred to as the self-prioritization effect (Kim, 2012; Rogers et al., 1977; Humphreys & Sui, 2016; Sui & Gu, 2017). For instance, in perceptual judgment tasks, self-relevant information (e.g., one’s own name or face) is processed more rapidly than other-relevant information (Keenan et al., 1999; Keyes & Brady, 2010; Ma & Han, 2010; Sui et al., 2006; Tong & Nakayama, 1999; Sui & Han, 2007). In memory domains, self-relevant materials are typically recalled more accurately than other-relevant materials (Conway, 2005; Conway & Pleydell-Pearce, 2000; Cunningham et al., 2008). Regarding attention, self-relevant information robustly captures attentional resources (Arnell et al., 1999; Brédart et al., 2006; Gronau et al., 2003; Pannese & Hirsch, 2010; Wolford & Morrison, 1980).

When self-relevant information acts as a target, individuals demonstrate an attentional advantage for self-relevant information compared to other-relevant information, characterized by enhanced response speed and accuracy. Furthermore, literature analysis reveals that this attentional advantage manifests not only in the spatial dimension but also in the temporal dimension. In the spatial dimension, Sui et al. (2006) required participants to judge facial orientation and found that orientation judgments for self-faces were faster than for familiar faces. Keyes and Brady (2010) similarly demonstrated significantly faster mouth-shape discrimination for self-faces compared to other-face conditions when participants judged lip postures. This attentional advantage effect also extends to transiently self-associated information (i.e., stimuli temporarily linked to the self through experimental conditioning). For example, Sui et al. (2012) associated neutral geometric shapes with distinct identities—a circle representing the self, a triangle representing a friend, and a square representing a stranger. Participants then performed a match-to-identity judgment task. Results revealed significantly faster reaction times in the self-associated condition compared to other-associated conditions (friend and stranger), and this effect withstood reductions in stimulus luminance contrast. In the temporal dimension, research using the rapid serial visual presentation (RSVP) paradigm has demonstrated that individuals typically fail to recognize words appearing within a few hundred milliseconds after a target word—a phenomenon known as the attentional blink. However, when the participant’s own name appears after the target word, identification of the self-name paradoxically occurs, indicating the attenuation of the attentional blink effect (Shapiro et al., 1997).

### 1.2. Attentional Capture Effect in Self-Relevant Information Processing

When self-relevant information acts as a distractor (i.e., non-target), it interferes with responses to targets, demonstrating attentional capture by self-relevant stimuli (Sui et al., 2014; Sui et al., 2015). This attentional capture effect specifically refers to the impact of self-relevant information on target processing when presented as distractors.

Empirical studies have demonstrated that self-names or self-faces continue to be selected and processed even when acting as distractors (Brédart et al., 2006; Moray, 1959; Wolford & Morrison, 1980). Similarly, Liu et al. (2014) adopted a global/local figure judgment task. By assessing individuals’ perceptual differences toward figures with distinct social significance (target vs. distractor levels), they measured the regulatory role of personally significant information on perceptual selection. Their findings revealed that, compared to other-associated figures, self-associated figures acting as distractors modulated the global precedence effect—a pattern consistently observed at both global and local processing levels. Numerous studies consistently demonstrate that the attentional capture of self-relevant information depends on its relevance to the task goal. When self-relevant information is pertinent to the current task goal—that is, when it has the potential to become a target—it exhibits a strong attentional capture effect (Gray et al., 2004; Sui et al., 2009; Sui et al., 2012).

However, when examining self-relevant information as a task-irrelevant stimulus, studies have yielded inconsistent conclusions. Some research has found that even when serving as an irrelevant stimulus, self-relevant information still exhibits a significant attentional capture advantage, thereby interfering with the performance of target tasks. Wolford and Morrison (1980) employed a flanker task to demonstrate that when participants performed parity judgments on two digits flanking a central distractor stimulus, reaction times slowed significantly when the distractor was the participant’s own name. Similarly, Brédart et al. (2006) found in a face–name interference paradigm that interference from one’s own face on judging a classmate’s name was significantly greater than interference from a classmate’s face on judging one’s own name. However, other studies have failed to replicate such findings. For instance, Bundesen et al. (1997) used a Stroop-like naming task and found that participants’ own name as a distractor did not compromise task accuracy. Kawahara and Yamada (2004) further controlled for potential confounding factors such as color saliency and name length and successfully replicated this pattern of results. Devue and Brédart (2008) found that the self-face did not possess a unique advantage in automatically capturing attention. Consequently, the researchers concluded that self-relevant information irrelevant to the current task does not elicit greater attentional capture effects. Liu et al. (2012) presented self-faces and familiar-other faces as task-irrelevant stimuli concurrently with target stimuli to examine how self-relevant information affects attentional selection processes when serving as non-target stimuli. Their findings failed to detect differential attentional capture effects between self-faces and familiar faces. Consequently, the researchers concluded that the distinctiveness of self-faces is primarily contingent upon task relevance.

Analysis of the above studies reveals that when self-relevant information appears as a task-relevant stimulus, it captures attention and interferes with target recognition. However, research has yet to reach a consensus regarding how task-irrelevant self-information (non-targets) impacts attentional processing. Elucidating this issue would significantly contribute to uncovering the cognitive mechanisms underlying self-relevant information processing. If self-relevant information as an irrelevant stimulus (non-target) still captures attention, this would suggest a stimulus-driven attentional processing mechanism for self-information. Conversely, if self-relevant information as an irrelevant stimulus (non-target) fails to capture attention, it indicates that the cognitive processing of self-information is not entirely automated but rather subject to modulation by cognitive factors such as strategic control or top-down goal sets.

### 1.3. Influence of Attentional Window on Attentional Capture

The size of the attentional window refers to the degree of attentional distribution during the initial scanning stage of information processing—that is, the scope of attentional focus. A large attentional window refers to the state where attention is distributed across the entire visual field, whereas a small attentional window refers to the state where attention is focused on a specific fixation point or a particular location. Based on this theoretical premise, researchers conducted a study and found that salient irrelevant stimuli can trigger attentional capture only when the attentional window is broadly diffused; if the attentional window is narrowed and focused on the central visual field, salient stimuli outside the attentional window are unable to capture attention. (Belopolsky & Theeuwes, 2010; Eriksen & St. James, 1986). The study by Bortolotti and Palumbo (2026) confirms that attention and perception are functionally and neurally independent processing mechanisms, providing the latest evidence for the modulatory effect of attention on perception. Another study, for instance, Belopolsky et al. (2007), instructed participants to search for a target letter that had an equal probability as other stimuli of being rendered in a salient color. Crucially, prior to the search task, participants were required to discriminate between global shapes (inducing distributed attention) and local shapes (inducing focused attention) to manipulate the size of the attentional window. The results demonstrated that under focused attention, salient-color stimuli—whether serving as targets or distractors—showed no significant difference in attentional capture compared to other stimuli. In contrast, during distributed attention, salient-color stimuli acting as distractors captured attention, and when serving as targets, elicited faster responses. These findings align with numerous established studies (Jonides & Yantis, 1988; Folk & Annett, 1994; Franconeri & Simons, 2003). Belopolsky and Theeuwes (2010) manipulated attentional window size by controlling fixation point presentations and configurations of peripheral stimuli forming virtual shapes. Under small attentional window conditions, fixation points consisted of randomly presented letter strings where participants first identified the central letter, only initiating target search when the letter was “K”, with no response otherwise. Under large (distributed) attentional window conditions, participants first discriminated shapes formed by peripheral virtual stimuli, responding only to circular configurations while withholding responses to square configurations. Crucially, the target stimulus was a green diamond, with distractor stimuli consisting of one red ring and the remaining distractors being green rings. This critical finding revealed that the red ring distractor failed to capture attention during small attentional windows but significantly captured attention during large attentional windows. Lu and Han (2009) demonstrated that attentional capture diminishes or even disappears when visual search tasks become more demanding (cf. Proulx & Egeth, 2006), whereas expanding the attentional window under such conditions reinstates attentional capture. Consequently, researchers propose that demanding tasks, brief target presentations, or fixed focal points constitute small attentional window states (Proulx & Egeth, 2006; Bacon & Egeth, 1994).

Keyes and Dlugokencka (2014) investigated self-relevant information as task-irrelevant stimuli (where self-information never served as targets). Their findings revealed that when self-faces and friend-faces appeared as task-irrelevant distractors concurrently with target names (in Experiment 1, an image of a distractor face appeared centrally—inside the focus of attention—behind a target name, with the faces either upright or inverted), self-faces failed to elicit stronger interference. Conversely, when faces were positioned outside attentional focus (in Experiment 2, distractor faces appeared peripherally—outside the focus of attention—in the left or right visual field, or bilaterally), self-faces significantly interfered with name recognition. Although the researchers did not discuss these findings from an attentional window perspective, the experimental manipulations correspond functionally to attentional window conditions based on its conceptual framework and operational parameters: Experiment 1 was equivalent to adopting a small attentional window paradigm (central distractor within focused attention), whereas Experiment 2 corresponded to a large attentional window paradigm (peripheral distractors requiring distributed attention). Consequently, rigorously manipulating attentional window parameters to investigate whether attentional window size modulates processing of task-irrelevant self-information will help clarify the controversy over whether self-related information automatically captures attention when it is irrelevant to the current goal.

### 1.4. Research Proposition

Based on the previous discussions, when it comes to the attentional processing mechanism underlying the attentional capture effect induced by self-related information, that is, whether this attentional capture is a bottom-up automatic capture of attention or regulated by top-down goal setting, there are currently two differing viewpoints in existing research. Some researchers have found that the attentional capture caused by self-related information is a completely automatic bottom-up attentional capture, independent of the influence of cognitive factors such as top-down goal setting, and is similar to stimulus-driven attentional capture (e.g., Humphreys & Sui, 2016; Sui et al., 2012; Sui et al., 2015; Alexopoulos et al., 2012). However, some other researchers have discovered that the attentional capture triggered by non-target self-related information is not bottom-up but top-down and is regulated by cognitive factors such as goal tasks (e.g., Devue & Brédart, 2008; Bundesen et al., 1997; Keyes & Dlugokencka, 2014).

In addition to attempting to clarify the aforementioned controversies, this study also found that when previous research explored the nature of attentional capture by self-related information, it primarily used reaction time as an indicator. However, reaction time results are a composite of multiple components, encompassing both the capture of attention by stimuli in the early stages of processing (attentional orientation) and the disengagement of attention from stimuli in the later stages of processing (attentional disengagement). Based on the literature review of the previous three parts, few researchers have separately discussed early attentional orienting and late attentional disengagement in the attentional capture process of self-related information. Separating attentional orienting from attentional disengagement holds significant importance because they occur at different stages of information processing and may be differentially influenced by cognitive factors such as goal setting or strategic attentional biases. Therefore, this study will discuss attentional orienting and disengagement separately.

Previous discussions have also suggested that the aforementioned controversy may stem from experimental procedures. Belopolsky and Theeuwes (2010) and Theeuwes (2010) found that when attention is dispersed across the entire visual field, the initial scanning stage of information processing is entirely stimulus-driven. In other words, whether a distractor is target-related (potentially becoming a target) or target-unrelated, as long as it exhibits higher saliency than the target, it will capture attention. Subsequently, the cognitive processing mechanism shifts to a top-down mode. When the attentional window is small, that is, when attention is focused on a specific point or when the target presentation time is brief, the initial stage of visual selection operates through top-down processing. Under these conditions, only target-related distractors capture attention, while target-unrelated distractors fail to capture attention. As mentioned earlier, although Keyes and Dlugokencka (2014) did not discuss the issue of self-related information from the perspective of the attention window in their study, the experimental manipulations in their two experiments were similar to scenarios involving different attention window sizes. Therefore, this study will employ more rigorous manipulations of the attention window to further investigate whether the attention window modulates the non-target processing of self-related information.

Based on the above, this study aims to explore the mechanisms of attentional capture (including attentional orientation and disengagement) by self-related information, as well as whether these mechanisms are modulated by the size of the attention window, by manipulating both the size of the attention window and the relevance of self-related information to the target. To achieve the aforementioned objectives, the entire study employs a learning-test paradigm. During the learning phase, participants are required to establish associations between colors and identity information. In the test phase, different attention window sizes and the relevance of self-related information to the target are manipulated. Additionally, a variant of the spatial cueing paradigm is utilized to isolate attentional orientation and attentional disengagement. This approach aims to investigate whether the attentional capture (including attentional orientation and disengagement) in the non-target processing of self-related information is fully automatic and whether it is modulated by the size of the attention window.

The specific operations are as follows: Three experiments were conducted in this study. Experiments 1 and 2 both used a small attentional window: self-related information was associated with the target in Experiment 1 but unrelated to the target in Experiment 2. In Experiment 3, we adopted a large attentional window while keeping self-related information irrelevant to the target. The specific experimental logic is as follows: Experiments 1 and 2 are designed to explore whether the attentional orientation and disengagement mechanisms in the non-target processing of self-related information are fully automatic (i.e., whether they are modulated by top-down goal setting) under the condition of a small attention window. Experiment 3 shifts to a large attention window condition to verify whether the results of Experiment 3 are consistent with those of Experiment 2 and to investigate whether there is a modulating effect of the attention window on the attentional orientation and disengagement mechanisms in the non-target processing of self-related information.

Based on previous research findings, the expectations of this study are as follows: (1) If the results of Experiment 1 and Experiment 2 are consistent, it indicates that under the condition of a small attention window, the non-target processing of self-related information is stimulus-driven. If the results of Experiment 1 and Experiment 2 are inconsistent, it suggests that under the condition of a small attention window, the non-target processing of self-related information is not fully automatic and can be modulated by top-down cognitive factors. (2) If the results of Experiment 3 are consistent with those of Experiment 2 (i.e., consistent outcomes under both large and small attention window conditions), it suggests that the attention window does not modulate the attentional orientation and disengagement processes in the non-target processing of self-related information. If the results of Experiment 3 differ from those of Experiment 2 (i.e., inconsistent outcomes under large and small attention window conditions), it indicates that the attention window can modulate the attentional orientation and disengagement processes in the non-target processing of self-related information.

## 2. Experiments 1 and 2

Mechanisms of attentional orientation and disengagement in the non-target processing of self-related information under the condition of a small attention window are examined.

Experimental objective: To investigate the mechanisms of attentional orientation and disengagement in the non-target processing of self-related information by manipulating the relevance of self-related information to the target under the condition of a small (concentrated) attention window.

Experimental procedure: In Experiments 1 and 2, during the learning phase, neutral stimuli (colors) were endowed with different identity meanings (self-related information/stranger information) through a color-identity association task (Vromen et al., 2016). Subsequently, in the test phase, participants were required to complete a visual search task. We drew on the classic experimental manipulation for inducing a small attentional window from Belopolsky and Theeuwes (2010). Specifically, participants were instructed to discriminate the shape of the fixation point to establish a small attentional window. Meanwhile, the relevance of self-related information to the target was manipulated to examine the mechanisms of attentional orientation and disengagement in the non-target processing of self-related information.

Experimental expectations: If the attentional capture by self-related information is driven by bottom-up stimulus factors, then regardless of whether the self-related information is relevant (Experiment 1) or irrelevant (Experiment 2) to the target, participants will exhibit rapid attentional orientation and delayed disengagement towards self-related information compared to stranger information. Conversely, if the attentional capture by self-related information is driven by top-down goal factors, then under the condition where self-related information is relevant to the target (Experiment 1), participants will demonstrate rapid attentional orientation and delayed disengagement towards self-related information compared to stranger information. However, under the condition where self-related information is irrelevant to the target (Experiment 2), there will be no significant differences in attentional orientation and disengagement between self-related information and stranger information.

### 2.1. Experiment 1: Self-Related Information Relevant to the Target

This experiment aims to investigate whether self-related information exhibits a significant attentional orientation advantage and delayed disengagement compared to stranger information when the self-related information is relevant to the target.

#### 2.1.1. Methods

(1) Participants

A total of 24 undergraduate students (12 males) were recruited through advertisements, with an average age of 19.54 ± 1.70 years. The sample size (*α* = 0.05, 1 − *β* = 0.80) was estimated using G*Power 3.1 software, assuming a moderate effect size (*ƒ* = 0.25), and the calculated required sample size was 24. In addition, sample size estimation was conducted based on the average partial eta squared ($\eta_{p}^{2}$ = 0.2685) reported in comparable studies, revealing that a maximum of 16 participants would be required for the present study (Bola et al., 2021). This criterion also applies to Experiment 2 and Experiment 3. Therefore, the sample size of this study meets the requirements. All participants had normal color vision (no color blindness or color weakness), normal or corrected-to-normal visual acuity, and were right-handed. They had not stayed up late or consumed alcohol the day before the experiment. After reading and understanding the informed consent form, they voluntarily participated in the experiment and were compensated with 25 yuan upon completion. This study was approved by the Academic Ethics and Morality Committee of the corresponding author’s institution.

(2) Experimental materials

The experimental materials were created with reference to the study by Anderson et al. (2016). The background for presenting the experimental materials was gray (RGB: 128, 128, 128), and the viewing distance was set at 60 cm. The size of the circular rings remained consistent between the learning phase and the test phase. The experimental materials consisted of circular rings in various colors, white lines with different orientations, as well as white and pink rectangular frames (2.9° × 2.9°). There were seven colors for the circular rings: red (RGB: 255, 0, 0), green (RGB: 0, 255, 0), orange (RGB: 255, 128, 0), yellow (RGB: 255, 255, 0), purple (RGB: 128, 0, 128), blue (RGB: 0, 128, 255), and cyan (RGB: 0, 255, 255). All the lines were white, oriented horizontally, vertically, or at a 45-degree or 135-degree incline. The experimental materials for the test phase also included white rectangular frames (RGB: 255, 255, 255), pink patches, and pink rectangular frames (RGB: 255, 128, 255). The experimental procedures were programmed, and the experimental data were recorded using the specialized software E-Prime 2.0.

(3) Experimental design

The learning phase (color-identity association task) employed a within-subjects design with two factors: 2 (identity information: self, stranger) × 2 (matching conditions: match, mismatch).

The test phase (visual search task) employed a single-factor within-subjects design. The independent variable was cue trial type, categorized into three types: target-cued trials, distractor-cued trials, and foil-cued trials. Among these, both target-cued and distractor-cued trials included three distractor conditions: self-related information distractors, stranger-related information distractors, and no-identity information distractors. Conversely, foil-cued trials only included two distractor conditions: self-related information distractors and stranger-related information distractors.

Dependent variable measures: Reaction time and accuracy rate.

(4) Experimental Procedure

Learning phase: color-identity association task.

**Figure 1:**
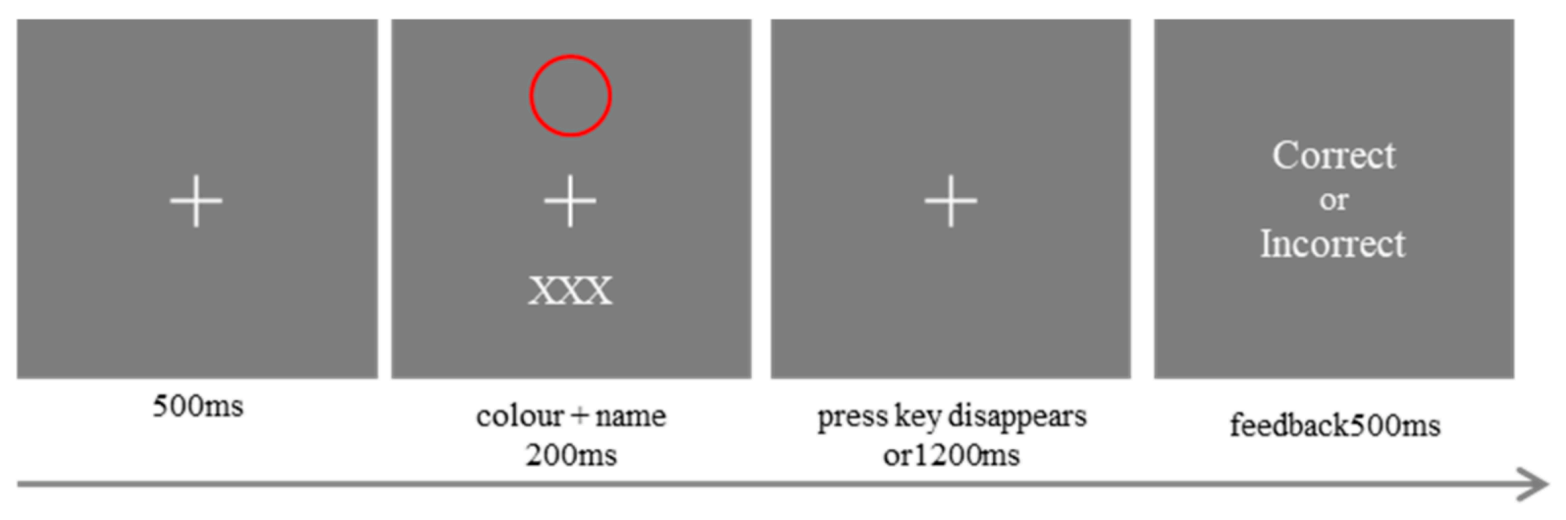
Experimental flowchart of the color-identity association task.

The process is illustrated in Figure 1:

① Participants were instructed that the red circular ring represented themselves, while the green circular ring represented a stranger (color assignments were counterbalanced across participants).

② A fixation cross “+” (2.8°) was displayed for 500 ms.

③ A red or green circular ring appeared randomly above the fixation cross, while the participant’s own name or a stranger’s name appeared randomly below it, both presented for 200 ms. The centers of the circular ring and the name were about 7° from the fixation point. Participants were required to judge whether the color of the ring matched the identity represented by the name. If they matched (e.g., red ring + self-name), participants pressed the “F” key; if they did not match (e.g., red ring + stranger’s name), participants pressed the “J” key (key assignments were counterbalanced across participants).

④ The response screen remained visible for 1200 ms or until a key press was made, after which feedback indicating correctness (correct/incorrect) was provided. The association phase consisted of 24 trials. Participants advanced to the test phase only if their accuracy rate reached 98% or higher; otherwise, they continued practicing.

Test phase: visual search task.

**Figure 2:**
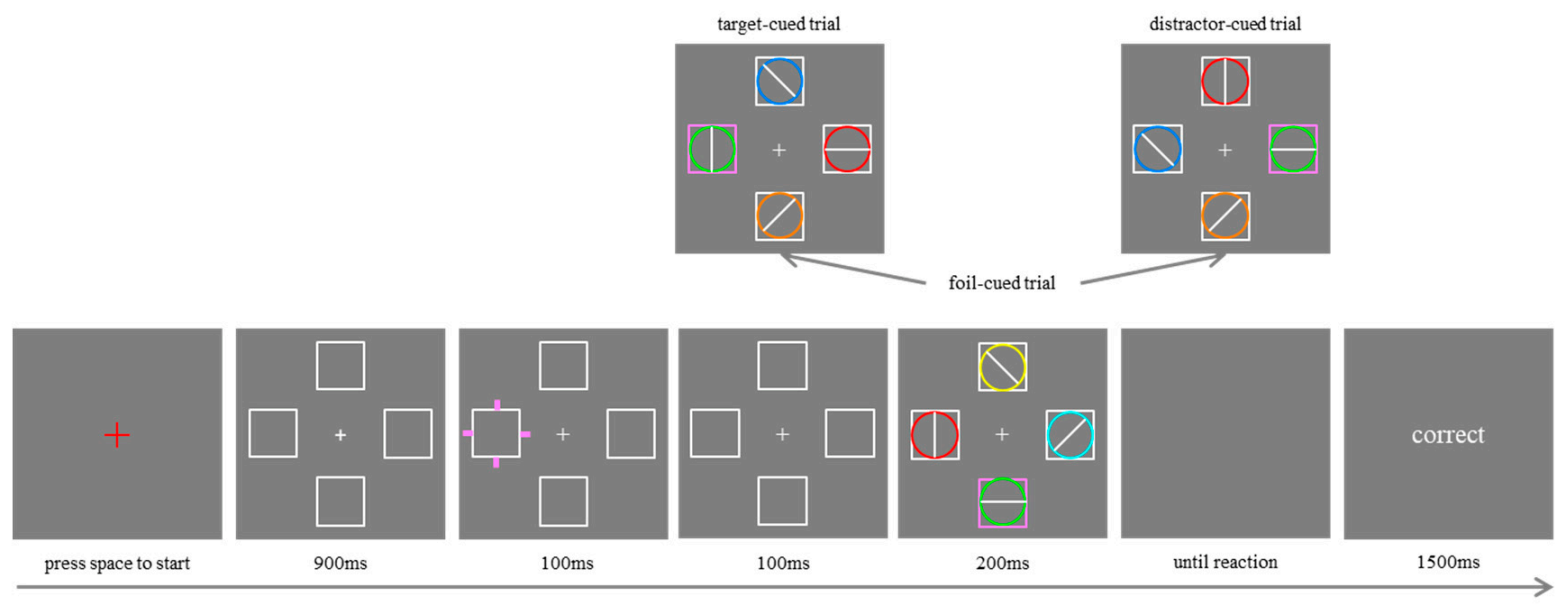
Experimental flowchart of the visual search task (Experiment 1).

The specific procedure is shown in Figure 2:

① A red fixation cross was presented at the center of the screen.

② After participants pressed the spacebar, the fixation cross turned white, and four white rectangular frames were simultaneously displayed for 900 ms. Each box was centered approximately 7° from the fixation point.

③ A cue (four pink patches flashing around one of the frames) was presented for 100 ms and then disappeared. The location of the cue might or might not match the subsequent location of the target circular ring (the position of the pink frame).

④ A fixation cross “+” (2.8°) and the four surrounding white rectangular frames were displayed for 100 ms.

⑤ The target search screen was presented for 200 ms, during which one of the frames turned pink. Participants were tasked with making a horizontal or vertical keypress response based on the orientation of the line inside the circular ring within the pink frame—pressing the “C” key for horizontal and the “M” key for vertical (key assignments were counterbalanced across participants). The circular ring inside the pink frame was either red or green (self-related information relevant to the target). The central fixation cross on the search screen had two possible forms: either a “+” or an “○”. If the central fixation cross was a “+”, participants made a keypress response; if it was an “○”, participants made no response.

⑥ After the search screen, an unlimited-time response screen appeared. Following the participant’s keypress, a feedback screen was displayed for 1500 ms, indicating whether the response was correct or incorrect, after which the next trial began.

Before the formal test phase, there were 48 practice trials. Participants could proceed to the formal experiment only if their accuracy rate reached 95% or higher. The formal experiment consisted of a total of 576 trials (288 response trials and 288 non-response trials), which were randomly divided into four equal-sized groups. Participants were allowed to take a short break after completing each group. Before the formal test phase, there were 48 practice trials. Participants could proceed to the formal experiment only if their accuracy rate reached 95% or higher. The formal experiment consisted of a total of 576 trials (288 response trials and 288 non-response trials), which were randomly divided into four equal-sized groups. Participants were allowed to take a short break after completing each group. The trial procedure was modeled after Vromen et al. (2016).

Taking response trials in the test phase as an example, when the cue location and the target location are the same, and the color of the circular ring inside the frame is either red or green, such trials are referred to as target-cued trials (72 trials in total). In these cases, if a circular ring of the other target color (red or green) appears inside a frame at a non-target location, it constitutes a distractor condition (either self-related information distractors or stranger-related information distractors). Conversely, if no such interfering ring appears, it is termed as a no-identity-information distractor condition.

When the cue location and the target location are different, and the color of the circular ring inside the frame at the cue location is a non-target color (i.e., a color other than red or green), such trials are termed distractor-cued trials (144 trials in total). In distractor-cued trials, if a circular ring of another target color (red or green) appears inside a frame at a non-target location (a non-pink frame), it constitutes a distractor condition (either self-related information distractors or stranger-related information distractors). If no such interfering ring appears, it is classified as a no-identity-information distractor condition.

When the cue location and the target location are different, and the circular rings within the frames at these two locations are colored red and green, respectively, such trials are referred to as foil-cued trials (72 trials in total). These trials only involve the distractor condition (either self-related information interference or stranger-related information interference).

#### 2.1.2. Results

Statistical analyses were conducted separately on the accuracy rates and reaction times data from the learning phase and the testing phase. The data from all participants were valid. The data analysis software used was SPSS 25.0. For the *p*-values of main effects and interactions that did not meet the sphericity assumption, the Greenhouse–Geisser method was employed for correction. After the analysis of variance (ANOVA), the *p*-values for post hoc multiple comparisons were corrected using the Bonferroni method.

(1) Learning Phase: Color-Identity Association Task

A 2 (identity information: self, stranger) × 2 (matching condition: match, mismatch) repeated-measures analysis of variance (ANOVA) was conducted on participants’ accuracy rates and reaction times during the learning phase.

In terms of reaction times, the main effect of identity information was significant, *F*(1, 23) = 7.685, *p* = 0.011, $\eta_{p}^{2}$ = 0.25, specifically demonstrating that reaction times for self-related information (365 ms) were significantly faster than those for stranger-related information (406 ms). The main effect of matching condition was not significant, *F*(1, 23) = 0.416, *p* = 0.526. The interaction between identity and matching condition was significant, *F*(1, 23) = 3.858, *p* = 0.048, $\eta_{p}^{2}$ = 0.167. Simple effects analysis revealed that under the matching condition, reaction times for self-related information (344 ms) were significantly faster than those for stranger-related information (417 ms) (*p* < 0.001). Under the mismatching condition, there was no significant difference in reaction times between self-related and stranger-related information, *F*(1, 23) = 0.435, *p* = 0.516. In terms of accuracy rates, no significant main effects or interaction effects were observed (all *p*s > 0.1). These findings suggest that participants successfully established robust associations between identity information and specific colored rings.

(2) Test Phase: Visual Search Task

The cueing effect refers to the differences in dependent variable measures (accuracy rates and reaction times) among target-cue trials, distractor-cue trials, and foil-cue trials.

Accuracy: A repeated-measures analysis of variance revealed a significant cueing effect, *F*(2, 46) = 3.86, *p* = 0.028, $\eta_{p}^{2}$ = 0.144. Specifically, the accuracy rate for target-cue trials (*M* = 96%, *SD* = 2%) was significantly higher than that for distractor-cue trials (*M* = 94%, *SD* = 4%, *p* = 0.043) and foil-cue trials (*M* = 94%, *SD* = 3%, *p* = 0.019). No significant difference was found between the accuracy rates of distractor-cue trials and foil-cue trials (*p* > 0.1).

Reaction times: A repeated-measures analysis of variance revealed a significant cueing effect, *F*(2, 46) = 9.471, *p* = 0.001, $\eta_{p}^{2}$ = 0.463. Specifically, the reaction time for target-cue trials (*M* = 614 ms, *SD* = 113 ms) was significantly shorter than that for distractor-cue trials (*M* = 647 ms, *SD* = 110 ms) and foil-cue trials (*M* = 663 ms, *SD* = 121 ms). Additionally, the reaction time for foil-cue trials was significantly longer than that for distractor-cue trials (*p* = 0.041).

Combining the results of these two indicators, it is evident that a significant cueing effect exists.

Orientation refers to the capture of attention by self-related distractions and stranger-related distractions when they appear at non-cued locations, primarily reflected in target-cue trials and distractor-cue trials.

Accuracy: In target-cue trials (including self-related information distractors, stranger-related information distractors, and no-identity-related information distractors, with within-subjects difference analysis conducted on these three conditions), a significant main effect of identity information was observed, *F*(2, 46) = 8.965, *p* = 0.001, $\eta_{p}^{2}$ = 0.449. Specifically, the accuracy rate under self-related information distractors (*M* = 94%, *SD* = 4%) was significantly lower than that under the no-identity-related information distractor condition (*M* = 98%, *SD* = 3%, *p* = 0.002). There was no significant difference in accuracy rates between self-related information distractors and stranger-related information distractors (*M* = 97%, *SD* = 4%, *p* = 0.139). Additionally, no significant difference was found between the accuracy rates of stranger-related information distractors and the no-identity-related information distractors condition (*p* = 0.375). In distractor-cue trials, a significant main effect of identity information was observed; *F*(2, 46) = 12.002, *p* < 0.001, $\eta_{p}^{2}$ = 0.343. Specifically, the accuracy rate under self-related information distractors (*M* = 91%, *SD* = 7%) was significantly lower than that under stranger-related information distractors (*M* = 96%, *SD* = 5%, *p* = 0.002) and the no-identity-related information distractors condition (*M* = 96%, *SD* = 4%, *p* = 0.004). No significant difference was found between the accuracy rates of stranger-related information distractors and the no-identity-related information distractors condition (*p* > 0.1).

Reaction times: In target-cue trials, a significant main effect of identity information was found; *F*(2, 46) = 8.132, *p* = 0.001, $\eta_{p}^{2}$ = 0.261. Specifically, the reaction time under self-related information distractors (*M* = 639 ms, *SD* = 130 ms) was significantly slower than that under stranger-related information distractors (*M* = 605 ms, *SD* = 110 ms, *p* = 0.035) and the no-identity-related information distractors condition (*M* = 598 ms, *SD* = 102 ms, *p* = 0.005). No significant difference was observed in reaction times between stranger-related information distractors and the no-identity-related information distractors condition (*p* > 0.1). In distractor-cue trials, a significant main effect of identity information was observed; *F*(2, 46) = 5.905, *p* = 0.005, $\eta_{p}^{2}$ = 0.204. Specifically, the reaction time under self-related information distractors (*M* = 663 ms, *SD* = 113 ms) was significantly longer than that under stranger-related information distractors (*M* = 643 ms, *SD* = 116 ms, *p* = 0.028) and the no-identity-related information distractors condition (*M* = 636 ms, *SD* = 107 ms, *p* = 0.014). No significant difference was found in reaction times between stranger-related information distractors and the no-identity-related information distractors condition (*p* > 0.1).

Combining the results of the aforementioned two indicators, it can be concluded that there exists attentional preferential orientation towards self-related information compared to stranger-related information.

Disengagement refers to the process where, when self-related information distractors and stranger-related information distractors appear at the cued location, participants need to shift their attention away from these self- or stranger-related interferences and redirect it toward the target location. This process is primarily reflected in foil-cue trials.

Accuracy: A paired-samples *t*-test revealed that the accuracy rate under self-related information distraction (*M* = 93%, *SD* = 5%) was significantly lower than that under stranger-related information distraction (*M* = 96%, *SD* = 3%), *t*(23) = −2.740, *p* = 0.012, Cohen’s *d* = 0.559.

Reaction Time: A paired-samples *t*-test showed that the reaction time under self-related information distraction (*M* = 677 ms, *SD* = 122 ms) was significantly longer than that under stranger-related information distraction (*M* = 650 ms, *SD* = 124 ms), *t*(23) = 3.204, *p* = 0.004, Cohen’s *d* = 0.654.

**Figure 3:**
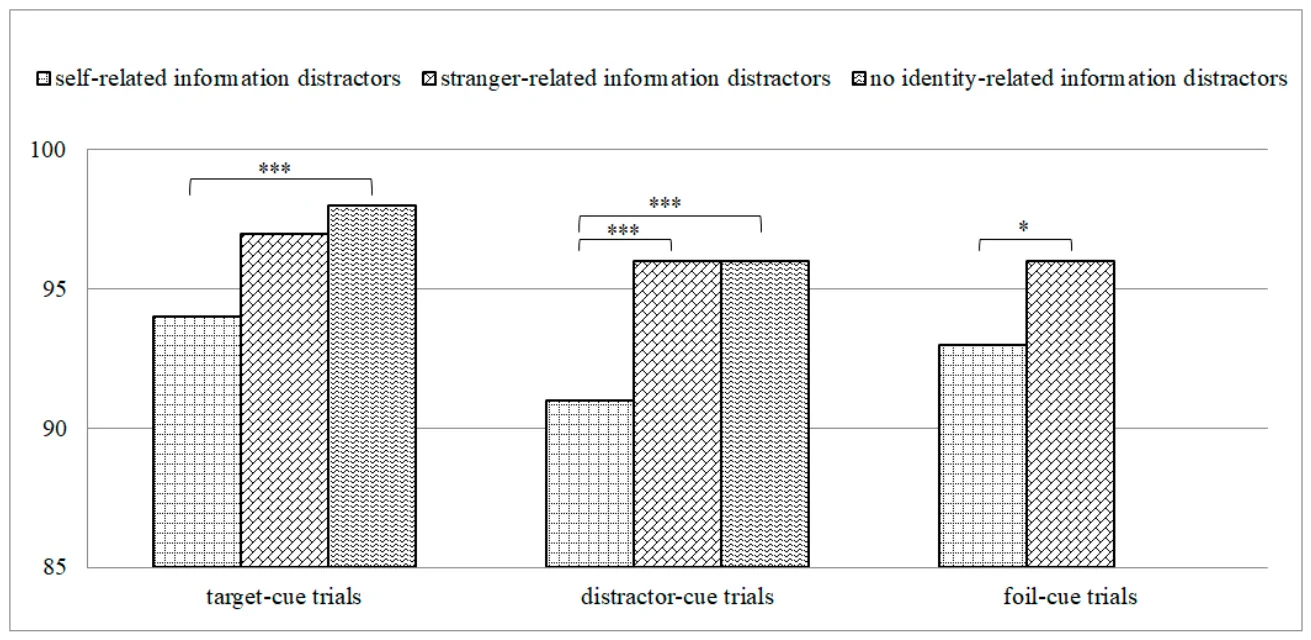
Accuracy of attentional orienting and disengagement in Experiment 1 (* *p* < 0.05, *** *p* < 0.001).

**Figure 4:**
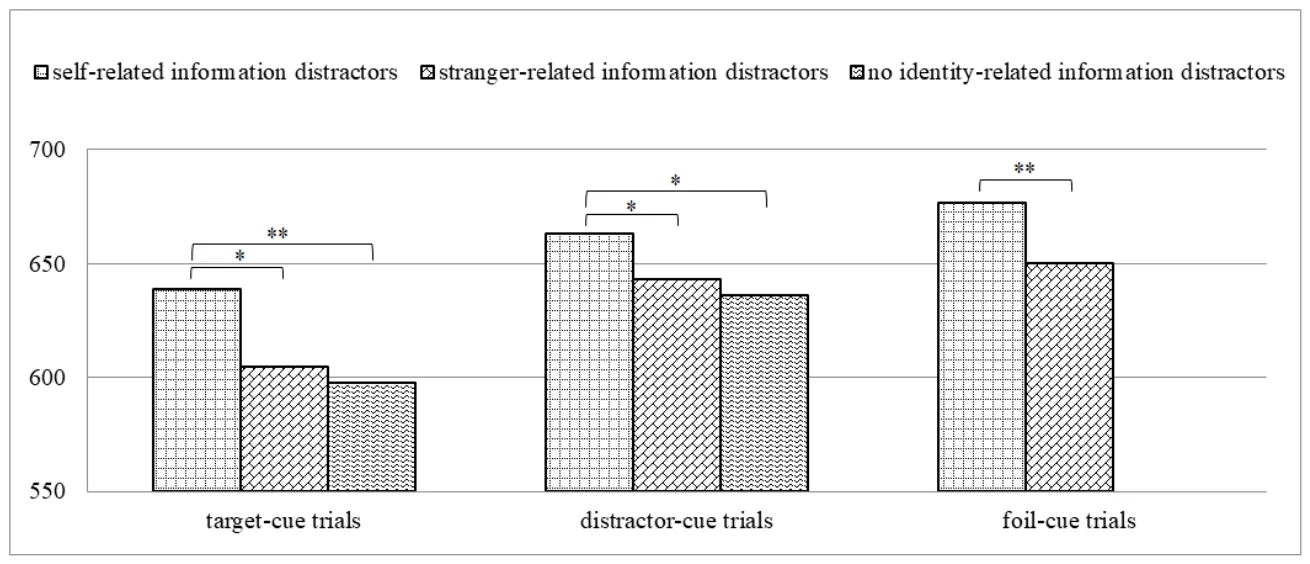
Reaction time of attentional orienting and disengagement in Experiment 1 (* *p* < 0.05, ** *p* < 0.01).

Combining these two indicators, it can be concluded that there exists a delay in attentional disengagement from self-related information compared to stranger-related information, Figure 3 and Figure 4.

#### 2.1.3. Discussion

The results of Experiment 1 indicate that during the learning phase, participants established associations between identity and specific colored rings. Specifically, participants exhibited significantly faster reaction times to rings colored in the hue representing self-related information compared to those representing stranger-related information. These findings are consistent with previous research (Sui et al., 2009; Sui et al., 2012). When the attentional window was narrow, during the test phase, self-related information exhibited attentional orienting priority and delayed disengagement compared to stranger-related information when associated with the target. Specifically, when the target ring appeared at the cued location, attention was already directed toward the target position, eliminating the need for further attentional shifts. Nevertheless, we still observed rapid attentional orienting toward self-related information. Meanwhile, when the target ring did not appear at the cued location, but self-related information was present there, delayed disengagement of attention from self-related information was also observed.

### 2.2. Experiment 2: Self-Related Information Is Irrelevant to the Target

The results of Experiment 1 demonstrated that when self-related information was relevant to the target, it facilitated faster attentional orienting and exhibited delayed disengagement compared to stranger-related information. Experiment 2 aimed to investigate the mechanisms of attentional orienting and disengagement during the non-target processing of self-related information when it was irrelevant to the target (specifically, during the associative learning phase, the targets were red or green rings, whereas in the visual search phase, the target square was pink, and the ring inside it was either orange or cyan). Previous studies have found that even when self-related information is irrelevant to the target, it can still automatically capture attention, supporting the claim that attentional capture by self-related information is fully automatic (e.g., Sui et al., 2015; Alexopoulos et al., 2012). If this viewpoint holds true, then the results of Experiment 2 should be consistent with those of Experiment 1. However, if the results of Experiment 2 diverge from those of Experiment 1, it would suggest that the processes of attentional orienting and disengagement during the non-target processing of self-related information are not fully automatic but are instead modulated by top-down goal-directed settings.

#### 2.2.1. Method

(1) Participants

A total of 24 undergraduate students (12 males) were recruited through advertisements, with an average age of 19.27 ± 1.08 years. Other participant characteristics were the same as those in Experiment 1.

(2) Experimental Materials

The experimental materials were the same as those in Experiment 1. The only difference was that in Experiment 2, the target ring inside the pink square was orange or cyan, while red or green appeared as one of the non-target colors.

(3) Experimental Design

The learning phase (color-identity association task) adopted a within-subjects design with two factors: 2 (identity information: self, stranger) × 2 (matching conditions: match, mismatch). The test phase (visual search task) employed a single-factor within-subjects experimental design, where the independent variable was the type of cued trial, categorized into target-cued trials, distractor-cued trials, foil-cued trials, and red/green-cued trials. Among them, target-cued trials, distractor-cued trials, and foil-cued trials each encompassed three conditions: self-related information distractors, stranger-related information distractors, and no-identity information distractors. In contrast, red/green-cued trials only included two conditions: self-related information distractors and stranger-related information distractors.

(4) Experimental Procedure

Learning Phase: The color-identity association task was identical in materials and procedures to the association phase of Experiment 1.

Test Phase: Visual search task.

**Figure 5:**
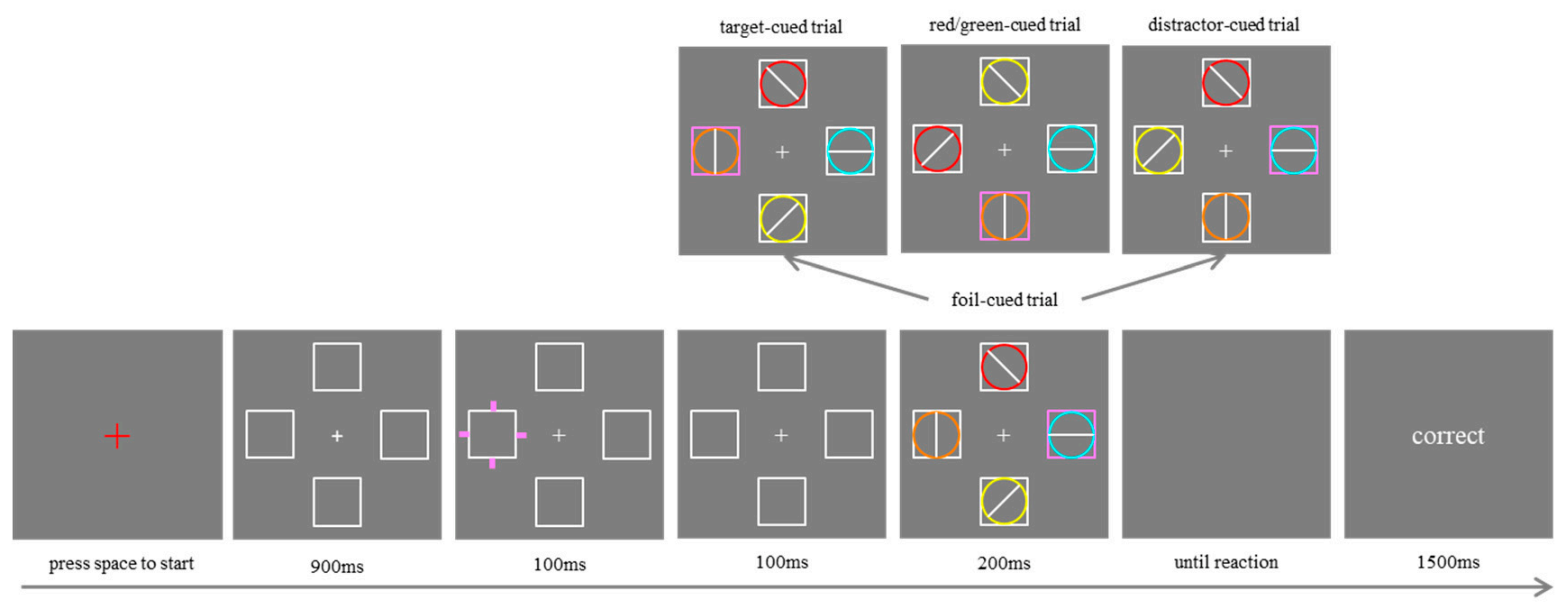
Experimental flowchart of the visual search task (Experiment 2).

The experimental procedure in the test phase was largely the same as that in Experiment 1, with the exception that the color of the ring inside the pink square at the target location was orange or cyan (self-related information is irrelevant to the target). The procedure for a single trial is illustrated in Figure 5. Again, we will use the response trial as an example.

Target-cued trials (72 trials in total): The cue location coincides with the target location. In such cases, if the color of the ring within the square at any other location is red or green, it constitutes a distractor condition (either self-related information distractors or stranger-related information distractors). If no red or green appears in the rings at other locations, it represents a no-identity information distractor condition.

Distractor-cued trials (72 trials in total): When the color of the ring within the square at the cue location is yellow or blue, if the color of the rings within the squares at other non-target locations is red or green, it constitutes a distractor condition (either self-related information distractors or stranger-related information distractors). If the colors of the rings within the squares at other non-target locations are neither red nor green, it represents a no-identity information distractor condition.

Foil-cued trials (72 trials in total): When different target colors (orange or cyan) appear in the squares at both the cue location and the target location, if the color of the rings within the squares at non-target locations is red or green, it constitutes a distractor condition (either self-related information distractors or stranger-related information distractors). If the colors of the rings within the squares at non-target locations are neither red nor green, it represents a no-identity information distractor condition.

Red/green-cued trials (72 trials in total): When the color of the ring within the square at the cue location is red or green, such trials only involve distractor conditions (either self-related information distractors or stranger-related information distractors).

#### 2.2.2. Result Analysis

Statistical analyses were conducted separately on the accuracy rates and reaction time data from the learning phase and the testing phase. The data from all participants were valid. The data analysis software employed was SPSS 25.0. For the main effects and interactions that did not meet the sphericity assumption, the *p*-values were corrected using the Greenhouse–Geisser method. After the analysis of variance (ANOVA), the *p*-values for post hoc multiple comparisons were corrected using the Bonferroni method.

(1) Color-identity association task

A 2 (identity information: self, stranger) × 2 (matching condition: match, mismatch) repeated-measures analysis of variance (ANOVA) was conducted on participants’ accuracy rates and reaction times during the association phase.

The results revealed that, in terms of reaction times: There was a significant main effect of identity information; *F*(1, 23) = 11.459, *p* = 0.003, $\eta_{p}^{2}$ = 0.333. Specifically, participants responded significantly faster to self-related information (401 ms) than to stranger-related information (458 ms). The main effect of matching condition was not significant; *F*(1, 23) = 0.239, *p* = 0.63. There was a significant interaction between identity information and matching condition; *F*(1, 23) = 11.304, *p* = 0.003, $\eta_{p}^{2}$ = 0.33. Simple effects analysis revealed that, under the matching condition, participants responded significantly faster to self-related information than to stranger-related information (*p* = 0.001). Under the mismatching condition, however, there was no significant difference in reaction times between self-related and stranger-related information; *F*(1, 23) = 0.308, *p* = 0.584. In terms of accuracy rates: There was a significant interaction between identity information and matching condition, *F*(1, 23) = 10.314, *p* = 0.004, $\eta_{p}^{2}$ = 0.31. Simple effects analysis revealed that the accuracy rate under the self-related information matching condition was significantly higher than that under the self-related information mismatching condition (*p* = 0.004). These findings suggest that participants successfully established associations between identity information and specific colored rings.

(2) Testing Phase: Visual Search Task

The cueing effect refers to the variations in dependent variable measures (accuracy rates and reaction times) across different trial types, namely target-cue trials, distractor-cue trials, foil-cue trials, and red/green-cue trials.

Accuracy rates: A repeated-measures analysis of variance revealed a significant cueing effect; *F*(3, 69) = 6.428, *p* = 0.001, $\eta_{p}^{2}$ = 0.218. Specifically, the accuracy rate for target-cue trials (*M* = 96%, *SD* = 3%) was significantly higher than that for distractor-cue trials (*M* = 93%, *SD* = 5%, *p* = 0.036), red/green-cue trials (*M* = 94%, *SD* = 4%, *p* = 0.019), and foil-cue trials (*M* = 93%, *SD* = 5%, *p* = 0.003). No significant differences were observed between the accuracy rates of distractor-cue trials and those of foil-cue trials or red/green-cue trials, nor between the accuracy rates of red/green-cue trials and foil-cue trials (*p*s > 0.1).

Reaction times: A repeated-measures analysis of variance revealed a significant cueing effect; *F*(3, 69) = 6.25, *p* = 0.003, $\eta_{p}^{2}$ = 0.472. Specifically, the reaction time for target-cue trials (*M* = 645 ms, *SD* = 103 ms) was significantly faster than that for distractor-cue trials (*M* = 696 ms, *SD* = 109 ms, *p* = 0.001), red/green-cue trials (*M* = 700 ms, *SD* = 112 ms, *p* = 0.001), and foil-cue trials (*M* = 702 ms, *SD* = 118 ms, *p* = 0.002). No significant differences were observed between the reaction times of distractor-cue trials and those of foil-cue trials or red/green-cue trials, nor between the reaction times of red/green-cue trials and foil-cue trials (*p*s > 0.01).

Based on the combined results of accuracy rates and reaction times, a significant cueing effect was demonstrated.

Orienting refers to the capture of attention by self-related information distractors and stranger-related information distractors when they appear at non-cued locations, encompassing target-cue trials, distractor-cue trials, and foil-cue trials.

Accuracy rates: The main effects of identity information were not significant in distractor-cue trials, *F*(2, 46) = 1.456, *p* = 0.255, BF_01_ = 2.2; in foil-cue trials, *F*(2, 46) = 1.041, *p* = 0.361, BF_01_ = 2.8; and in target-cue trials, *F*(2, 46) = 0.229, *p* = 0.769, BF_01_ = 4.5.

Reaction times: The main effects of identity information were not significant in distractor-cue trials, *F*(2, 46) = 0.059, *p* = 0.942, BF_01_ = 10.2; in foil-cue trials, *F*(2, 46) = 0.633, *p* = 0.535, BF_01_ = 3.7; and in target-cue trials, *F*(2, 46) = 2.523, *p* = 0.091, BF_10_ = 1.8.

The combined results above indicate that there are no significant differences between self-related information and stranger-related information in terms of attentional orienting.

Disengagement refers to the process where, when self-related information distractors or stranger-related information distractors appear at the cued location, participants need to shift their attention away from the self-related or stranger-related information and redirect it to the target location. This process is manifested in red/green-cue trials.

Accuracy: Paired-samples *t*-tests revealed no significant difference in accuracy rates between self-related information distractors (*M* = 94%, *SD* = 5%) and stranger-related information distractors (*M* = 93%, *SD* = 5%), *t*(23) = 0.562, *p* = 0.579, BF_01_ = 2.5.

Reaction times: Paired-samples *t*-tests showed no significant difference in reaction times between self-related information distractors (*M* = 702 ms, *SD* = 115 ms) and stranger-related information distractors (*M* = 699 ms, *SD* = 118 ms), *t*(23) = 0.401, *p* = 0.692, BF_01_ = 3.1.

**Figure 6:**
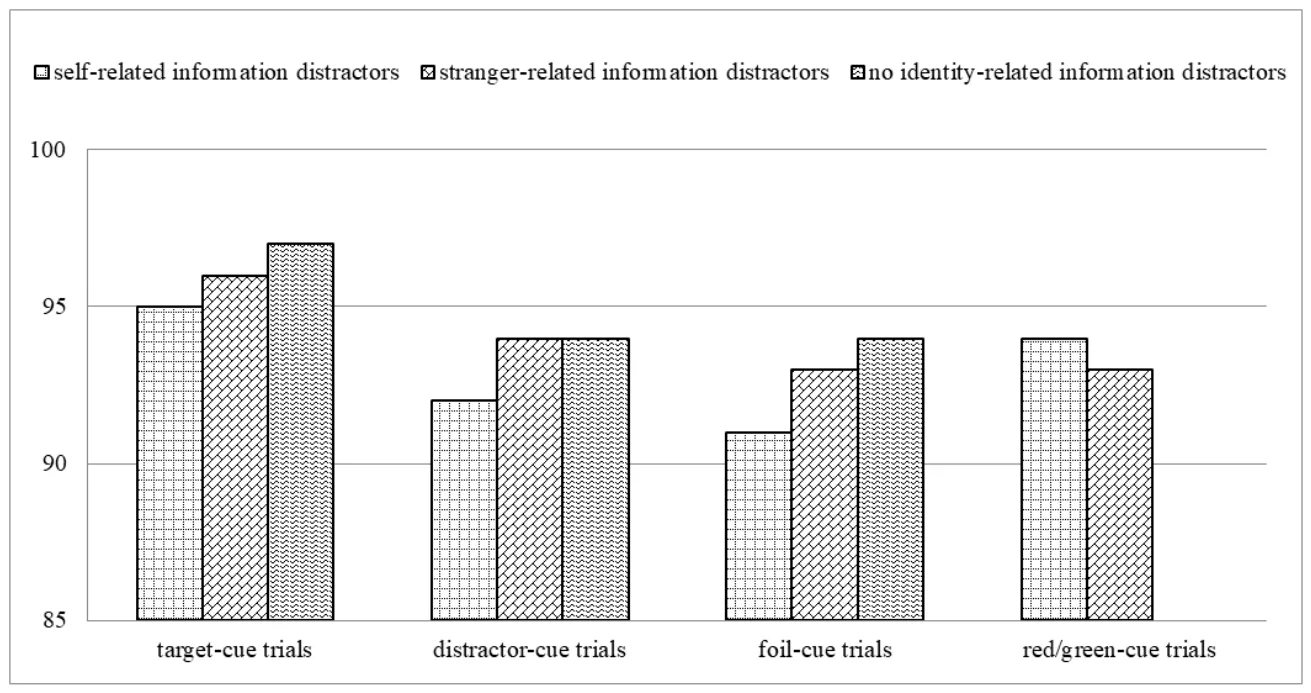
Accuracy of attentional orienting and disengagement in Experiment 2.

**Figure 7:**
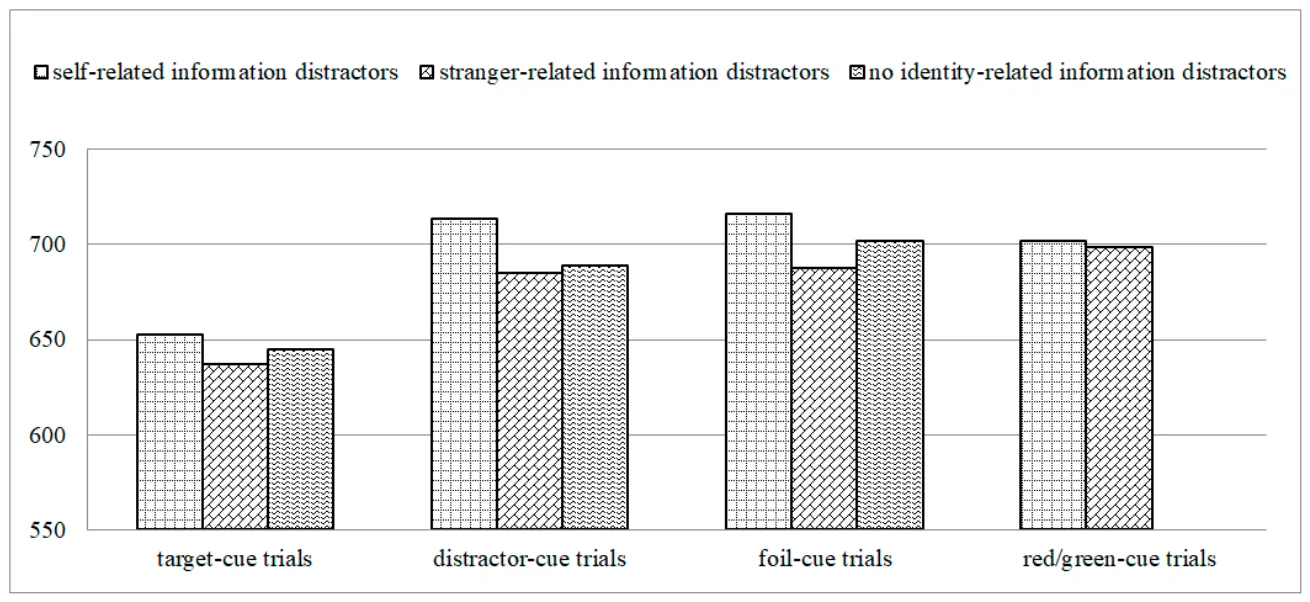
Reaction time of attentional orienting and disengagement in Experiment 2.

The combined results above indicate that there are no significant differences between self-related information and stranger-related information in terms of attentional disengagement, Figure 6 and Figure 7.

#### 2.2.3. Discussion

The results of Experiment 2 indicate that during the learning phase, participants established associations between identity and specific colored rings. Specifically, participants responded significantly faster to the colored ring representing self-related information compared to the one representing stranger-related information, which is consistent with findings from previous studies (Sui et al., 2009; Sui et al., 2012). During the test phase, under the condition of a small attentional window, when self-related information was irrelevant to the target, there were no significant differences between self-related information and stranger-related information in both the attentional orienting and disengagement stages. This result aligns with the inferences drawn based on the attentional window theory (Theeuwes, 2010). According to the attentional window theory, when the attentional window is small, visual selection operates through top-down processing, where only stimuli relevant to the target capture attention, while stimuli unrelated to the target do not automatically attract attention. This explains the non-significant differences in the results of Experiment 2. By integrating the findings from Experiment 2 with those of Experiment 1, we can conclude that the attentional capture process (including both attentional orienting and disengagement) for self-related information during non-target processing is not entirely automatic but is modulated by top-down goal-directed settings.

## 3. Experiment 3

Here, we examine the attentional orienting and disengagement mechanisms in the non-target processing of self-relevant information under large window conditions.

Experimental Objective: To verify whether, under conditions of a large (dispersed) attentional window, the attentional capture (including attentional orienting and disengagement) process in the non-target processing of self-relevant information is modulated by top-down goal set.

Experimental Procedure: After the color-identity association task, participants were required to complete a visual search task. The large (dispersed) attentional window condition was manipulated by increasing the presentation duration of the search screen while asking participants to discriminate the shapes of peripheral virtual figures. Under this condition, we investigated the attentional orienting and disengagement mechanisms in the non-target processing of self-related information when such information was irrelevant to the task goals.

Experimental Expectations: The results of Experiment 1 and 2 indicate that when the attentional window is small (focused), top-down goal set can modulate the attentional orienting and disengagement in the non-target processing of self-related information. If the attentional window can regulate the attentional orienting and disengagement processes in self-related information processing, it can be inferred that when the attentional window is large (dispersed), there will be differences in the attentional orienting and disengagement mechanisms of non-target processing of self-related information compared to when the attentional window is small. In other words, different results from those in Experiment 2 are expected to be found in Experiment 3. If the attentional window fails to modulate the attentional orienting and disengagement mechanisms in the non-target processing of self-related information, then consistent results with those of Experiment 2 are expected to be observed in Experiment 3.

### 3.1. Experiment 3: Self-Related Information Is Irrelevant to the Task Goal Under Large Attentional Window Conditions

#### 3.1.1. Method

(1) Participants

A total of 29 undergraduate students (14 males) were recruited through advertisements, with an average age of 22.13 ± 1.98 years. Other participant characteristics were the same as those in Experiment 1.

(2) Experimental Materials

The experimental materials were largely the same as those in Experiment 2. The difference was that the trials with a fixation point of “○” in Experiment 2 were removed. In Experiment 3, the virtual shape formed by the four peripheral figures could be either a rhombus or a square, and these two shapes were presented randomly.

(3) Experimental Design

The learning phase (the color-identity association task) employed a within-subjects design with a 2 (identity information: self, stranger) × 2 (matching condition: match, mismatch), which was the same as in Experiment 1 and Experiment 2. The test phase (visual search task) employed a single-factor within-subjects experimental design, with the independent variable being the type of cue trial. There were four types of cue trials: target-cue trials, distractor-cue trials, foil-cue trials, and red/green-cue trials. Among them, target-cue trials, distractor-cue trials, and foil-cue trials each included three conditions: self-related information distractors, stranger-related information distractors, and no-identity information distractors. In contrast, red/green-cue trials only encompassed two conditions: self-related information distractors and stranger-related information distractors.

Dependent variable measures: reaction time and accuracy rate.

(4) Experimental Procedure

Learning Phase: The color-identity association task followed the exact same procedure as the association phase in Experiment 1 and Experiment 2.

Test Phase: Visual search task.

**Figure 8:**
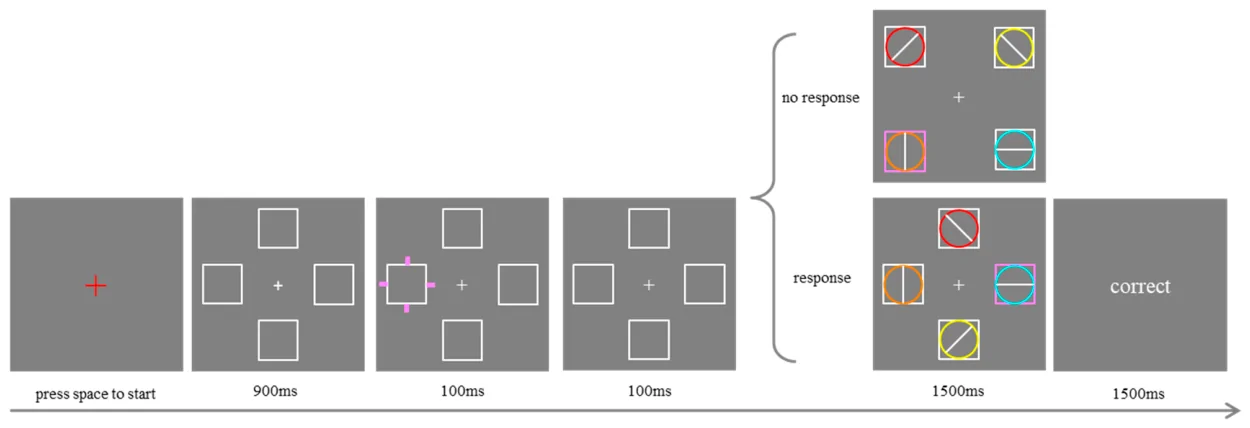
Experimental flowchart of the visual search task (Experiment 3).

The experimental procedure during the test phase was largely similar to that of Experiment 2, with the key difference being that participants were first required to judge the shape of the virtual figure formed by the four outer square frames on the search screen. They were to respond if the shape was a diamond and withhold response if it was a square. Both response trials and non-response trials were interspersed with a “+” fixation point. The procedure for a single trial is illustrated in Figure 8.

#### 3.1.2. Results

Statistical analyses were conducted separately on the accuracy rates and reaction times from the learning phase and the test phase. The data from all participants were valid. The data analysis was performed using SPSS 25.0. For main effects and interactions that did not meet the sphericity assumption, the *p* values were corrected using the Greenhouse–Geisser method. Following the analysis of variance (ANOVA), *p* values for post hoc multiple comparisons were adjusted using the Bonferroni correction.

(1) Learning Phase: Color-Identity Association Task

A 2 (identity information: self, stranger) × 2 (matching condition: match, mismatch) repeated-measures analysis of variance (ANOVA) was conducted on the accuracy rates and reaction times of participants during the learning phase. The results revealed that for reaction times, there was a significant main effect of identity information; *F*(1, 28) = 28.959, *p* < 0.001, $\eta_{p}^{2}$ = 0.508. Specifically, participants responded significantly faster to self-related information than to stranger-related information. The main effect of the matching condition was not significant; *F*(1, 28) = 0.077, *p* = 0.783. However, there was a significant interaction between identity information and matching condition; *F*(1, 28) = 35.026, *p* < 0.001, $\eta_{p}^{2}$ = 0.556. Simple effects analysis revealed that under the matching condition, the reaction time for self-related information (311 ms) was significantly faster than that for stranger-related information (391 ms) (*p* < 0.001). Under the non-matching condition, there was no significant difference in reaction times between self-related and stranger-related information (*p* = 0.333). In terms of accuracy rates, there was a significant main effect of matching condition, *F*(1, 28) = 10.046, *p* = 0.004, $\eta_{p}^{2}$ = 0.264, with participants showing significantly higher accuracy for self-related information compared to stranger-related information. Additionally, there was a significant interaction between identity and matching condition; *F*(1, 28) = 22.535, *p* < 0.001, $\eta_{p}^{2}$ = 0.446. Simple effects analysis revealed that under the matching condition, the accuracy rate for self-related information was significantly higher than that for stranger-related information (*p* < 0.001). Under the non-matching condition, there was no significant difference in accuracy rates between self-related and stranger-related information (*p* = 0.068). These results indicate that participants successfully established associations between identity information and specific colored rings.

(2) Test phase: Visual Search Task

The differences in dependent variable measures (accuracy rate and reaction time) across target-cue trials, distractor-cue trials, foil-cue trials, and red/green-cue trials are referred to as the cueing effect.

Accuracy: A repeated-measures analysis of variance (ANOVA) revealed a significant cueing effect; *F*(3, 84) = 14.568, *p* < 0.001, $\eta_{p}^{2}$ = 0.342. Specifically, the accuracy rate for target-cue trials (*M* = 95%, *SD* = 2%) was significantly higher than that for distractor-cue trials (*M* = 92%, *SD* = 2%, *p* = 0.018), red/green-cue trials (*M* = 91%, *SD* = 3%, *p* = 0.002), and foil-cue trials (*M* = 90%, *SD* = 3%, *p* < 0.001). The accuracy rate for distractor-cue trials was significantly higher than that for foil-cue trials (*p* = 0.010) but did not differ significantly from that for red/green-cue trials (*p* = 0.507). Additionally, there was no significant difference in accuracy rates between red/green-cue trials and foil-cue trials (*p* = 0.413).

Reaction Time: A repeated-measures analysis of variance (ANOVA) revealed a significant cueing effect; *F*(3, 84) = 7.667, *p* = 0.001, $\eta_{p}^{2}$ = 0.500. Specifically, the reaction time for target-cue trials (*M* = 785 ms, *SD* = 128 ms) was significantly shorter than that for distractor-cue trials (*M* = 826 ms, *SD* = 120 ms, *p* = 0.001), red/green-cue trials (*M* = 840 ms, *SD* = 130 ms, *p* < 0.001), and foil-cue trials (*M* = 819 ms, *SD* = 117 ms, *p* = 0.005). There were no significant differences in reaction times between distractor-cue trials and foil-cue trials, nor between distractor-cue trials and red/green-cue trials. Similarly, there was no significant difference in reaction times between red/green-cue trials and foil-cue trials (*p*s > 0.1).

Taking together the results of both accuracy rate and reaction time, it can be concluded that a significant cueing effect is evident.

Orientation refers to the capture of attention when self-related information and stranger-related information appear at non-cued locations, primarily manifested in target-cue trials, distractor-cue trials, and foil-cue trials.

Accuracy: In target-cue trials, a significant main effect of identity information was observed; *F*(2, 56) = 19.267, *p* < 0.001, $\eta_{p}^{2}$ = 0.408. Specifically, the accuracy rate under self-related information distractors (*M* = 92%, *SD* = 3%) was significantly lower than that under stranger-related information distractors (*M* = 95%, *SD* = 4%, *p* = 0.008) and that under the no-identity-information distractors condition (*M* = 97%, *SD* = 3%, *p* < 0.001). Additionally, the accuracy rate under stranger-related information distractors was significantly lower than that under the no-identity-information distractors condition (*p* = 0.025). In distractor-cue trials, a significant main effect of identity information was found; *F*(2, 56) = 17.726, *p* < 0.001, $\eta_{p}^{2}$ = 0.388. Specifically, the accuracy rate under self-related information distractors (*M* = 89%, *SD* = 4%) did not differ significantly from that under stranger-related information distractors (*M* = 92%, *SD* = 4%, *p* = 0.143) but was significantly lower than that under the no-identity-information interference condition (*M* = 96%, *SD* = 4%, *p* < 0.001). Additionally, the accuracy rate under stranger-related information distractors was significantly lower than that under the no-identity-information interference condition (*p* = 0.001). In foil-cue trials, a significant main effect of identity information was observed; *F*(2, 56) = 44.883, *p* < 0.001, $\eta_{p}^{2}$ = 0.616. Specifically, the accuracy rate under self-related information distractors (*M* = 87%, *SD* = 4%) was significantly lower than that under stranger-related information distractors (*M* = 91%, *SD* = 4%, *p* < 0.001) and that under the no-identity-information distractors condition (*M* = 93%, *SD* = 4%, *p* < 0.001). Additionally, the accuracy rate under stranger-related information distractors was significantly lower than that under the no-identity-information distractors condition (*p* = 0.028).

Reaction Time: In target-cue trials, a significant main effect of identity information was found; *F*(2, 56) = 11.065, *p* < 0.001, $\eta_{p}^{2}$ = 0.450. Specifically, the reaction time under self-related information distractors (*M* = 809 ms, *SD* = 142 ms) was significantly longer than that under stranger-related information distractors (*M* = 772 ms, *SD* = 126 ms, *p* < 0.001) and that under the no-identity-information interference condition (*M* = 773 ms, *SD* = 122 ms, *p* = 0.002). There was no significant difference in reaction times between stranger-related information distractors and the no-identity-information interference condition (*p* = 0.956). In distractor-cue trials, a significant main effect of identity information was also observed; *F*(2, 56) = 13.817, *p* < 0.001, $\eta_{p}^{2}$ = 0.506. Specifically, the reaction time under self-related information distractors (*M* = 846 ms, *SD* = 130 ms) was significantly longer than that under stranger-related information distractors (*M* = 807 ms, *SD* = 113 ms, *p* < 0.001) and that under the no-identity-information interference condition (*M* = 822 ms, SD = 121 ms, *p* < 0.001). There was no significant difference in reaction times between stranger-related information distractors and the no-identity-information interference condition (*p* = 0.124). In foil-cue trials, a significant main effect of identity information was evident; *F*(2, 56) = 17.01, *p* < 0.001, $\eta_{p}^{2}$ = 0.378. Specifically, the reaction time under self-related information distractors (*M* = 841 ms, *SD* = 126 ms) was significantly longer than that under stranger-related information distractors (*M* = 805 ms, *SD* = 119 ms, *p* < 0.001) and that under the no-identity-information interference condition (*M* = 812 ms, *SD* = 112 ms, *p* = 0.001). There was no significant difference in reaction times between stranger-related information distractors and the no-identity-information interference condition (*p* = 0.717).

The aforementioned results of accuracy rates and reaction times indicate that self-related information demonstrates preferential attentional orientation compared to stranger-related information.

Disengagement refers to the process where, when self-related information and stranger-related information appear at the cued locations, attention needs to shift away from either self-related or stranger-related information towards the target location. This process is primarily manifested in red/green-cue trials.

Accuracy: Paired-samples *t*-tests revealed that the accuracy rate under self-related information distractors (*M* = 90%, *SD* = 3%) was significantly lower than that under stranger-related information distractors; (*M* = 93%, *SD* = 4%), *t*(28) = –5.572, *p* < 0.001, Cohen’s *d* = 1.035.

Reaction Time: Paired-samples *t*-tests showed that the reaction time under self-related information distractors (*M* = 858 ms, *SD* = 143 ms) was significantly longer than that under stranger-related information distractors; (*M* = 822 ms, *SD* = 120 ms), *t*(28) = 4.618, *p* < 0.001, Cohen’s *d* = 0.858.

**Figure 9:**
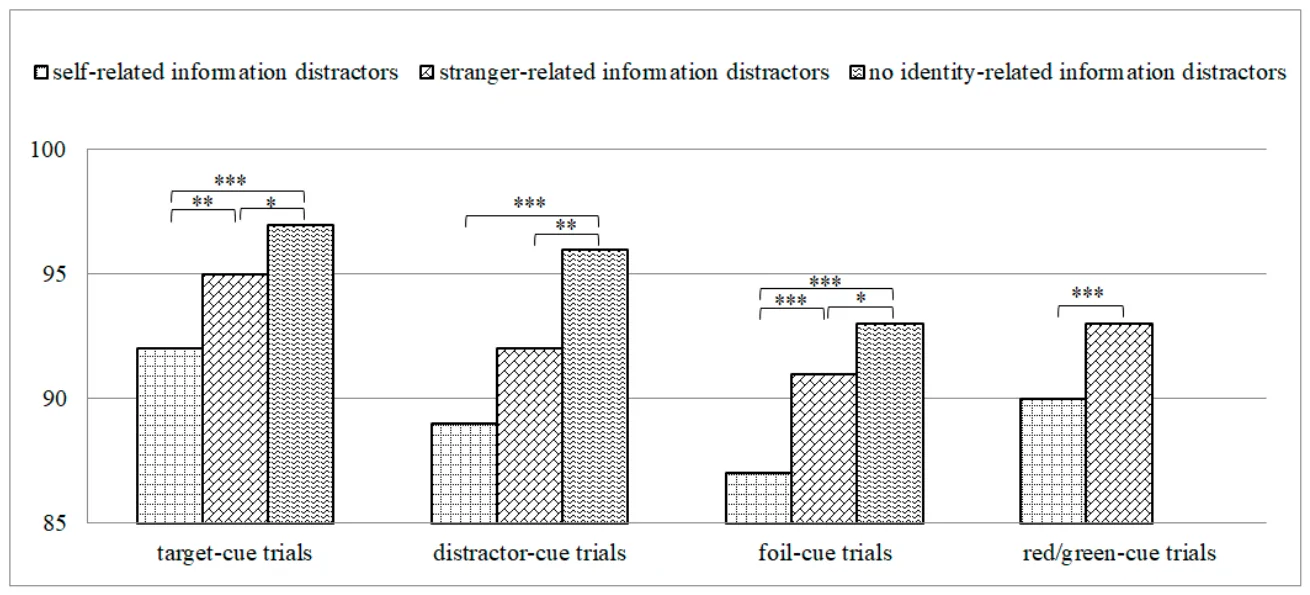
Accuracy of attentional orienting and disengagement in Experiment 3 (* *p* < 0.05, ** *p* < 0.01, *** *p* < 0.001).

**Figure 10:**
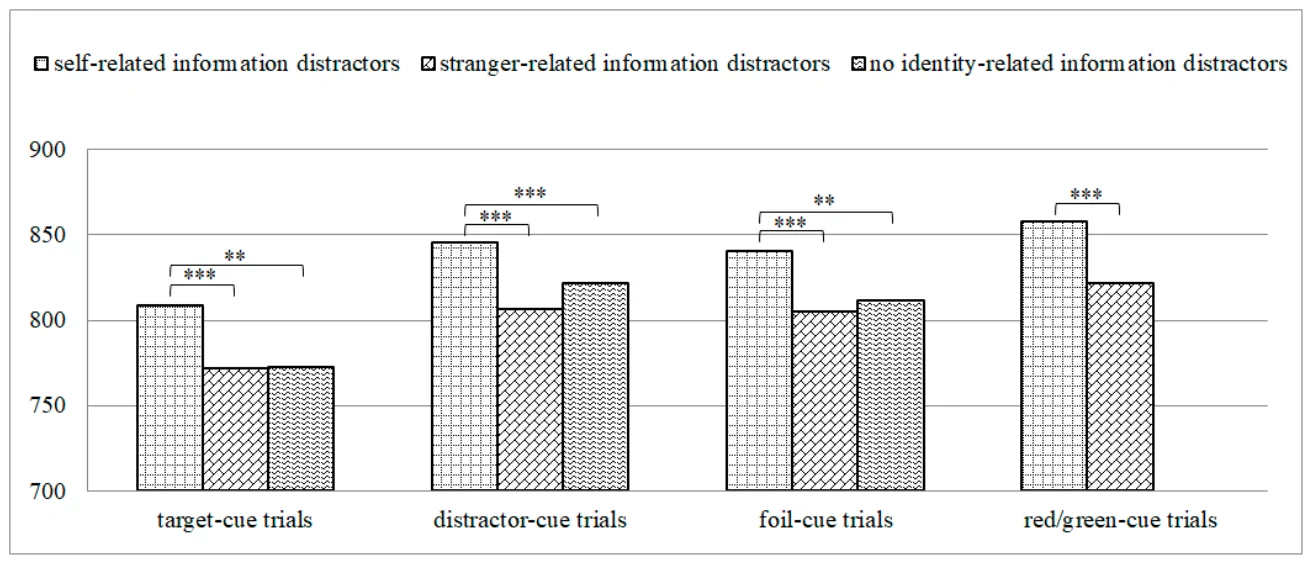
Reaction time of attentional orienting and disengagement in Experiment 3 (** *p* < 0.01, *** *p* < 0.001).

The aforementioned results of accuracy rates and reaction times indicate that self-related information exhibits delayed attentional disengagement compared to stranger-related information, Figure 9 and Figure 10.

#### 3.1.3. Discussion

The results of Experiment 3 indicate that during the learning phase, participants established associations between identity and specific colored rings. Specifically, participants responded significantly faster to colored rings representing self-related information compared to those representing stranger-related information, which is consistent with findings from previous studies (Sui et al., 2009; Sui et al., 2012). During the test phase, under the condition of a large attentional window, when the target ring appeared at the cued location, attention was already positioned at the target ring, eliminating the need for further attentional shifts. Nevertheless, the experiment still observed rapid attentional orientation toward self-related information. Conversely, when self-related information appeared at the cued location, participants had to disengage their attention from the cued position and shift it to the target position. The experimental results revealed delayed disengagement of attention from self-related information in this scenario. The above results demonstrate that under conditions with a large (dispersed) attentional window, even when self-related information is irrelevant to the target, it can still preferentially capture attention (including both attentional orientation and disengagement) compared to stranger-related information.

Based on the above analysis, it can be seen that the results of Experiment 3 are inconsistent with those of Experiment 2. This verifies the hypothesis that the attentional window modulates the mechanisms of attentional orientation and disengagement in the non-target processing of self-related information.

## 4. General Discussion

This study employed a modified version of the spatial cueing paradigm to investigate the attentional capture mechanisms (including attentional orientation and disengagement) underlying the non-target processing of self-related information, as well as the moderating effect of attentional window size, by manipulating whether self-related information was relevant to the target and the dimensions of the attentional window. The experimental results are as follows: During the learning phase, participants in all three experiments (Experiment 1, Experiment 2, and Experiment 3) successfully established associations between self-related information and specific colored rings. During the test phase, under the condition of a small attentional window, when self-related information was relevant to the target, it exhibited preferential attentional orientation and prolonged disengagement time compared to stranger-related information (Experiment 1). When self-related information was irrelevant to the target, no significant differences were observed between self-related and stranger-related information in terms of attentional orientation or disengagement (Experiment 2). Under the condition of a large attentional window, when self-related information was irrelevant to the target, it still demonstrated preferential attentional orientation and prolonged disengagement time compared to stranger-related information (Experiment 3).

First, it is highly noteworthy that during the association-building phase across all three experiments, we employed a between-subjects counterbalancing method to establish connections between differently colored rings and identity categories. This approach effectively controlled for potential confounding effects arising from variations in color salience, thereby eliminating the influence of stimulus prominence on the experimental results.

Moreover, in Experiment 1, regardless of whether it was in distractor-cue trials or target-cue trials, the difference in reaction times between self-related information and stranger-related information was statistically significant, whereas the difference in reaction times between stranger-related information and neutral conditions was not significant. The results indicate that only colors associated with self-related information demonstrated a more pronounced ability to capture attention, whereas the performance of stranger-related information was similar to that of neutral colors (colors not designated as targets during the association phase). This finding, to a certain extent, rules out the influence of learning effects and aligns with previous research results (Sui et al., 2009; Sui et al., 2012). The accuracy results also largely support this conclusion. Although in target-cue trials, there was a significant difference in accuracy between self-related information and the neutral condition, while no significant difference was observed between self-related information and stranger-related information, there was also no significant difference in accuracy between stranger-related information and the neutral condition. These findings suggest that even if learning played a role, its impact was minimal. Additionally, in distractor-cue trials, there were significant differences in accuracy between self-related information and both stranger-related information as well as the neutral condition, while no significant difference in accuracy was found between stranger-related information and the neutral condition. This result suggests that there was no influence of learning.

Secondly, during the test phase under the condition of a small attentional window, attentional capture was observed in Experiment 1 (where self-related information automatically captured attention), whereas no such attentional capture occurred in Experiment 2 (where self-related information failed to automatically capture attention). Therefore, based on the results of Experiments 1 and 2, it can be concluded that when the attentional window is small, top-down goal setting can modulate the attentional orientation and disengagement mechanisms involved in the non-target processing of self-related information. This conclusion supports the notion that the processing of self-related information as irrelevant stimuli is not entirely automatic, and this has been validated in both the early stage of processing, where stimuli capture attention (attentional orientation), and the late stage of processing, where attention disengages from stimuli (attentional disengagement).

Although the results of Experiment 1 and Experiment 2 support the conclusion that the non-target processing of self-related information is not entirely automatic, it is worth noting that these two experiments differ from previous studies in the following two key aspects. (1) Different research paradigms: In addition to manipulating the small attentional window, Experiments 1 and 2 also dissected the attentional capture process, analyzing the processing stages by dividing them into two phases: attentional orientation and attentional disengagement. (2) The manipulations in Experiments 1 and 2 incorporated the attentional window theory. According to this theory, based on the stimulus presentation durations and task difficulty levels in Experiments 1 and 2, these experiments fall under the category of having a small attentional window. Building upon these two differences and integrating the attentional window theory, this study further designed Experiment 3 to explore the reliability and stability of the conclusions drawn from Experiments 1 and 2. The specific operational setup was as follows: In both Experiment 2 and Experiment 3, self-related information was irrelevant to the target. However, unlike Experiment 2, Experiment 3 was conducted under conditions of a large attentional window. According to the study findings, the results of Experiment 3 were inconsistent with those of Experiment 2. The results of Experiment 3 demonstrated that, under conditions of a large attentional window, even though self-related information was irrelevant to the target, it could still automatically capture attention. Therefore, based on the results of Experiments 2 and 3, it can be concluded that the attentional window can modulate the attentional capture mechanism (including attentional orientation and attentional disengagement) involved in the non-target processing of self-related information.

Based on the results of the three experiments, it is evident that this series of experiments effectively elucidates a key issue: the previous debates surrounding the cognitive processing mechanisms of attentional capture by self-related information may stem from the fact that different experiments were conducted under attentional windows of varying sizes, leading to a multitude of inconsistent conclusions. According to the attentional window theory, when the attentional window is small, attentional load is high, and only stimuli relevant to the current goal are capable of capturing attention. Conversely, when the attentional window is large, meaning attention is dispersed across the entire visual field, cognitive load is lower, and individuals possess sufficient cognitive resources. In this scenario, they tend to prioritize objects with higher stimulus salience within the visual field. Given that self-related information holds greater psychological prominence and biological significance, it rapidly captures attention and exhibits delayed disengagement (Campanella et al., 2002).

Finally, these three series of experiments reveal that external environmental factors, such as the attentional window, can influence individuals’ processing modes of self-related information. This suggests that individuals exhibit flexibility in processing self-related information, rather than relying solely on either stimulus-driven or goal-driven processing. Instead, they adopt diverse and adaptable processing strategies based on the context, reflecting their ability to effectively adapt and respond to environmental changes.

This study employed a paradigm to distinguish between attentional orientation and disengagement. However, one inherent limitation of this paradigm arises when self-related information is relevant to the target. During the disengagement phase, since the cue location is marked by a green ring (associated with strangers during the learning phase) and the target location is indicated by a red ring (associated with the self during the learning phase), the speed of disengagement might be influenced by the differing levels of identity attractiveness represented by the rings in the target location. Specifically, the red ring, representing the self, may possess greater attractiveness compared to the green ring, representing strangers. However, based on the experimental results, the impact of such differing attractiveness does not appear to be significant. This conclusion is drawn from a comparison between the disengagement phases in the paradigms where self-related information is relevant to the target and where it is irrelevant. In both cases, during the disengagement phase, the cue location consistently features a green ring. Under the relevant condition, the target location displays a red ring (representing the self), whereas under the irrelevant condition, it shows a neutral-colored ring. If there were indeed notable differences in attractiveness, we would anticipate significantly shorter reaction times under the relevant condition compared to the irrelevant one. However, our findings indicate no significant difference in reaction times between the two conditions. This suggests that the disengagement process is not influenced by differences in the attractiveness of the target location but rather by the varying ease of disengaging from rings of different identities at the cue location. In addition, although the longer duration of the search display in Experiment 3 compared with Experiments 1 and 2 was designed to establish the large attentional window condition, such differences still exist. Therefore, follow-up studies could adopt alternative paradigms to further verify the robustness of this conclusion.

## 5. Conclusions

This study employed a modified version of the spatial cueing paradigm to investigate the attentional orientation and attentional disengagement mechanisms in the non-target processing of self-related information, as well as to explore whether the attentional window could serve as a modulating factor. The conclusions drawn are as follows: Under conditions of a small attentional window, the non-target processing of self-related information (attentional capture) is not entirely automatic; instead, both attentional orientation and attentional disengagement are subject to modulation by top-down goal-directed settings. Additionally, the series of results indicate that the size of the attentional window can modulate the attentional orientation and disengagement mechanisms in the non-target processing of self-related information. These findings reveal that although self-related information possesses significant salience, it is still subject to modulation by top-down goal-directed settings. External environmental factors, such as the attentional window, also influence individuals’ processing modes of self-related information. This suggests that individuals exhibit flexibility in processing self-related information, not adhering solely to either stimulus-driven or goal-driven processing. Instead, they adopt a variety of flexible processing strategies based on the context, reflecting their ability to effectively adapt and respond to environmental changes.

## Data Availability

Data are available from the corresponding author upon reasonable request. The data are not publicly available due to privacy and ethical considerations.
